# Reported Non–Substance-Related Mental Health Disorders Among Persons Who Died of Drug Overdose — United States, 2022

**DOI:** 10.15585/mmwr.mm7334a3

**Published:** 2024-08-29

**Authors:** Amanda T. Dinwiddie, Stephanie Gupta, Christine L. Mattson, Julie O’Donnell, Puja Seth

**Affiliations:** 1 Division of Overdose Prevention, National Center for Injury Prevention and Control, CDC.

**Summary**

**What is already known about this topic?**

During 2022, nearly 108,000 persons died of drug overdose in the United States. Persons with substance use disorders and non–substance-related mental health disorders, which frequently co-occur, are at increased risk for overdose.

**What is added by this report?**

In 2022, 22% of persons who died of drug overdose had a non–substance-related mental health disorder. The most common disorders were depressive (13%) and anxiety (9%). Approximately one quarter of decedents with a non–substance-related mental health disorder had at least one recent potential opportunity for intervention (e.g., current treatment for substance use disorders or recent emergency department visit).

**What are the implications for public health practice?**

Implementing evidence-based screening for substance use and mental health disorders during potential intervention opportunities and expanding efforts to integrate care for these disorders could improve mental health and reduce overdoses.

## Abstract

Drug overdose deaths remain a public health crisis in the United States; nearly 107,000 and nearly 108,000 deaths occurred in 2021 and 2022, respectively. Persons with mental health conditions are at increased risk for overdose. In addition, substance use disorders and non–substance-related mental health disorders (MHDs) frequently co-occur. Using data from CDC’s State Unintentional Drug Overdose Reporting System, this report describes characteristics of persons in 43 states and the District of Columbia who died of unintentional or undetermined intent drug overdose and had any MHD. In 2022, 21.9% of persons who died of drug overdose had a reported MHD. Using the *Diagnostic and Statistical Manual of Mental Disorders, Fifth Edition* criteria, the most frequently reported MHDs were depressive (12.9%), anxiety (9.4%), and bipolar (5.9%) disorders. Overall, approximately 80% of overdose deaths involved opioids, primarily illegally manufactured fentanyls. Higher proportions of deaths among decedents with an MHD involved antidepressants (9.7%) and benzodiazepines (15.3%) compared with those without an MHD (3.3% and 8.5%, respectively). Nearly one quarter of decedents with an MHD had at least one recent potential opportunity for intervention (e.g., approximately one in 10 decedents were undergoing substance use disorder treatment, and one in 10 visited an emergency department or urgent care facility within 1 month of death). Expanding efforts to identify and address co-occurring mental health and substance use disorders (e.g., integrated screening and treatment) and strengthen treatment retention and harm reduction services could save lives.

## Introduction

 Drug overdose deaths remain a public health crisis in the United States; nearly 107,000 and nearly 108,000 deaths occurred in 2021 and 2022, respectively.* Persons with mental health conditions are at increased risk for nonfatal and fatal overdose (*1*). The *Diagnostic and Statistical Manual of Mental Disorders, Fifth Edition* (DSM-5) defines mental health conditions, including both substance use disorders (SUDs) and non–substance-related mental health disorders (MHDs) (e.g., depressive, anxiety, and bipolar disorders). SUDs and MHDs commonly co-occur as both have shared risk factors and can influence each other (e.g., persons with certain MHDs might use substances for coping).^†^ In 2022, 23.1% of U.S. adults reported an MHD in the past year, and 8.4% had co-occurring MHDs and SUDs.^§^ Although mental health is an important consideration for overdose risk, characteristics of persons who died of overdose and had any non–substance-related MHD have not been widely studied.

## Methods

### Data Source

Data from CDC’s State Unintentional Drug Overdose Reporting System (SUDORS)^¶^ were analyzed to identify evidence and type of MHD among persons who died of unintentional or undetermined intent drug overdose during 2022. Jurisdictions participating in SUDORS entered data from death certificates, postmortem toxicology reports, and medical examiner and coroner reports into a web-based system.

### Identification of Mental Health Disorders

Jurisdictions used available source documents to identify MHDs (e.g., documentation of a diagnosis in the medical examiner or coroner report); non–substance-related MHD type** was selected from a drop-down menu or written into a free-text box.^††^ For this analysis, two independent analysts reviewed and categorized text box MHD entries according to the DSM-5; a licensed clinical psychologist confirmed categorizations and resolved discrepancies.

### Data Analysis

For deaths with and without evidence of MHD, decedent demographics and selected overdose circumstances were examined among 43 states and the District of Columbia (jurisdictions)^§§^ with complete medical examiner or coroner data^¶¶^ for the first and second halves of 2022; in addition, drug involvement was examined among 43 jurisdictions that also had complete data on drugs causing death during 2022.*** The following recent potential intervention opportunities to prevent overdose within 1 month of death were also examined: release from an institutional setting (i.e., prison or jail, residential treatment facility, or psychiatric hospital), treatment for SUD, emergency department or urgent care visit for any reason, or nonfatal overdose. Because the data represent a census of deaths in included jurisdictions, Pearson chi-square tests were used to compare characteristics of decedents with and without an MHD; for variables with multiple categories, pairwise comparisons were conducted if the global p-value was <0.05. Analyses were performed using SAS software (version 9.4; SAS Institute). This activity was reviewed by CDC, deemed not research, and was conducted consistent with applicable federal law and CDC policy.^†††^

## Results

### Frequency of Mental Health Disorders

During 2022, among 63,424 unintentional and undetermined intent drug overdose deaths across 44 jurisdictions, 21.9% of decedents had any reported non–substance-related MHD (Table 1). By DSM-5 criteria, the most common disorders were depressive (12.9%), anxiety (9.4%), and bipolar (5.9%).

**TABLE 1:** Reported non–substance-related mental health disorders* among persons who died of unintentional or undetermined intent drug overdose — State Unintentional Drug Overdose Reporting System, United States,^†^ 2022

**Non–substance-related mental health disorders** ^§^	**No. of decedents**	**% of all decedents n = 63,424**	**% of decedents with any reported mental health disorder n = 13,897**
Any mental health disorder	13,897	21.9	100.0
Depressive disorders^¶^	8,189	12.9	58.9
Anxiety disorders**	5,983	9.4	43.1
Bipolar and related disorders^††^	3,728	5.9	26.8
Schizophrenia spectrum and other psychotic disorders^§§^	1,988	3.1	14.3
Trauma- and stressor-related disorders^¶¶^	1,712	2.7	12.3
Neurodevelopmental disorders***	1,363	2.1	9.8
Other mental health disorders^†††^	889	1.4	6.4
Unspecified mental health disorders^§§§^	361	0.6	2.6

**Abbreviation:** SUDORS = State Unintentional Drug Overdose Reporting System.

* Evidence of mental health disorders was obtained from available source documents (e.g., medical records or witness report of a diagnosis in the medical examiner or coroner report) and categorized by *Diagnostic and Statistical Manual of Mental Disorders, Fifth Edition* classification.

^†^ The District of Columbia and the following 35 states reported deaths from the full jurisdiction: Alaska, Arizona, Arkansas, Colorado, Connecticut, Delaware, Georgia, Hawaii, Iowa, Kansas, Kentucky, Maine, Maryland, Massachusetts, Michigan, Minnesota, Mississippi, Montana, Nebraska, Nevada, New Hampshire, New Jersey, New Mexico, North Carolina, Ohio, Oklahoma, Oregon, Rhode Island, South Dakota, Tennessee, Utah, Vermont, Virginia, West Virginia, and Wisconsin. The following eight states reported deaths from counties that accounted for ≥75% of drug overdose deaths in the respective state, per SUDORS funding requirements: Alabama, Illinois, Indiana, Louisiana, Missouri, New York, Pennsylvania, and Washington. These 44 jurisdictions were included because death certificates and medical examiner or coroner reports were available for ≥75% of deaths during either 6-month reporting period (January–June or July–December 2022). Analyses were restricted to deaths with an available medical examiner or coroner report (92.3% of all deaths included).

^§^ Categories are not mutually exclusive. Decedents might have had more than one reported mental health disorder.

^¶^ Includes depression (except manic depression, which was included in the category for bipolar and related disorders), dysthymia, and other depressive disorders.

** Includes agoraphobia, anxiety, claustrophobia, panic disorder, and social phobia.

^††^ Includes bipolar and manic depression.

^§§^ Includes delusional disorder, paranoid disorder, psychoactive disorder, psychotic disorder, schizoaffective disorder, schizophrenia, schizophreniform disorder, and schizotypal disorder.

^¶¶^ Includes adjustment disorder, grief reaction, stress disorder, and posttraumatic stress disorder.

*** Includes attention-deficit/hyperactivity disorder, autism spectrum, borderline intellectual functioning, developmental disorder, dyslexia, learning disability/disorder, tic disorder, and Tourette's disorder.

^†††^ Includes other disorders that did not fit into a specific category listed as personality disorders; mood disorders; sleep-wake disorders; obsessive-compulsive and related disorders; feeding and eating disorders; neurocognitive disorders; disruptive, impulse-control, or conduct disorders; and somatic symptom and related disorders.

^§§§^ Includes broader unspecified results for mental condition, disorder, or illness, and psychiatric disease or disorder.

### Demographics and Selected Circumstances

Compared with those without a reported MHD, higher percentages of decedents with any reported MHD were female (40.0% versus 25.9%) and non-Hispanic White (White) (71.1% versus 61.4%), and lower percentages were non-Hispanic Black or African American (Black) (15.9% versus 24.8%) and Hispanic or Latino (Hispanic) (8.8% versus 10.3%) (Table 2). A higher percentage of decedents with an MHD had a known history of opioid use or misuse compared with those without an MHD (42.4% versus 29.8%).

**TABLE 2:** Demographic characteristics, select circumstances, and drug involvement among persons who died of unintentional or undetermined intent drug overdose, by non–substance-related mental health disorder status* — State Unintentional Drug Overdose Reporting System, United States,^†^ 2022

Characteristic	Overdose deaths, no. (%)		
	Total N = 63,424	With any reported mental health disorder n = 13,897	Without reported mental health disorder n = 49,527
**Sex** ^§^			
Female**^¶^**	**18,386 (29.0)**	5,553 (40.0)	12,833 (25.9)
Male**^¶^**	**45,036 (71.0)**	8,343 (60.0)	36,693 (74.1)
**Age group, yrs^§^**			
<15**^¶^**	**193 (0.3)**	17 (0.1)	176 (0.4)
15–24**^¶^**	**3,675 (5.8)**	901 (6.5)	2,774 (5.6)
25–34	**13,624 (21.5)**	3,047 (21.9)	10,577 (21.4)
35–44	**16,770 (26.4)**	3,762 (27.1)	13,008 (26.3)
45–54	**13,428 (21.2)**	2,894 (20.8)	10,534 (21.3)
55–64**^¶^**	**12,036 (19.0)**	2,553 (18.4)	9,483 (19.1)
≥65**^¶^**	**3,694 (5.8)**	723 (5.2)	2,971 (6.0)
**Race and ethnicity^§^**			
American Indian or Alaska Native, non-Hispanic	**1,087 (1.7)**	263 (1.9)	824 (1.7)
Asian, non-Hispanic	**406 (0.6)**	97 (0.7)	309 (0.6)
Black or African American, non-Hispanic**^¶^**	**14,351 (22.9)**	2,190 (15.9)	12,161 (24.8)
Native Hawaiian or Pacific Islander, non-Hispanic	**63 (0.1)**	9 (0.1)	54 (0.1)
White, non-Hispanic**^¶^**	**39,837 (63.5)**	9,780 (71.1)	30,057 (61.4)
Hispanic or Latino**^¶^**	**6,258 (10.0)**	1,218 (8.8)	5,040 (10.3)
Multiple races, non-Hispanic**^¶^**	**735 (1.2)**	206 (1.5)	529 (1.1)
**Select circumstances**			
Potential bystander present**^¶^**^,^**	**26,955 (42.5)**	6,485 (46.7)	20,470 (41.3)
Fatal drug use witnessed**^¶^**	**5,094 (8.0)**	1,046 (7.5)	4,048 (8.2)
Naloxone administered**^¶^**	**14,147 (22.3)**	3,191 (23.0)	10,956 (22.1)
Ever treated for SUD**^¶^**	**7,845 (12.4)**	2,974 (21.4)	4,871 (9.8)
History of opioid use or misuse**^¶^**	**20,651 (32.6)**	5,897 (42.4)	14,754 (29.8)
**Drugs involved** ^††^			
Antidepressants**^¶^**	**2,961 (4.7)**	1,334 (9.7)	1,627 (3.3)
Benzodiazepines**^¶^**	**6,294 (10.0)**	2,113 (15.3)	4,181 (8.5)
Any opioid**^¶^**	**51,578 (82.2)**	11,216 (81.4)	40,362 (82.4)
Heroin**^¶^**^,§§^	**4,645 (7.4)**	946 (6.9)	3,699 (7.6)
IMFs**^¶^**^,¶¶^	**47,188 (75.2)**	9,807 (71.2)	37,381 (76.3)
Prescription opioids**^¶^**^,^***	**7,890 (12.6)**	2,204 (16.0)	5,686 (11.6)
Any stimulant**^¶^**	**36,102 (57.5)**	7,206 (52.3)	28,896 (59.0)
Cocaine**^¶^**	**19,174 (30.6)**	3,639 (26.4)	15,535 (31.7)
Methamphetamine**^¶^**	**18,324 (29.2)**	3,724 (27.0)	14,600 (29.8)
Prescription stimulants**^¶^**^,§§§^	**922 (1.5)**	326 (2.4)	596 (1.2)

**Abbreviations:** IMFs= illegally manufactured fentanyl and fentanyl analogs; SUD = substance use disorder; SUDORS = State Unintentional Drug Overdose Reporting System.

* Evidence of mental health disorders was obtained from available source documents (e.g., medical records or witness report of a diagnosis in the medical examiner or coroner report) and categorized by *Diagnostic and Statistical Manual of Mental Disorders, Fifth Edition* classification.

^†^ The District of Columbia and the following 35 states reported deaths from the full jurisdiction: Alaska, Arizona, Arkansas, Colorado, Connecticut, Delaware, Georgia, Hawaii, Iowa, Kansas, Kentucky, Maine, Maryland, Massachusetts, Michigan, Minnesota, Mississippi, Montana, Nebraska, Nevada, New Hampshire, New Jersey, New Mexico, North Carolina, Ohio, Oklahoma, Oregon, Rhode Island, South Dakota, Tennessee, Utah, Vermont, Virginia, West Virginia, and Wisconsin. The following eight states reported deaths from counties that accounted for ≥75% of drug overdose deaths in the respective state, per SUDORS funding requirements: Alabama, Illinois, Indiana, Louisiana, Missouri, New York, Pennsylvania, and Washington. These 44 jurisdictions were included because death certificates and medical examiner or coroner reports were available for ≥75% of deaths during either 6-month reporting period (January–June or July–December 2022). Analyses were restricted to deaths with an available medical examiner or coroner report (92.3% of all deaths included).

^§^ Missing values were excluded from calculations of percentages. Percentages might not sum to 100% because of rounding.

^¶^ Pearson chi-square was p<0.05, indicating a statistically significant difference between decedents with and without a mental health disorder.

** Potential bystander is defined in the footnotes section of the SUDORS Data Dashboard. https://www.cdc.gov/overdose-prevention/data-research/facts-stats/sudors-dashboard-fatal-overdose-data.html

^††^ A drug was considered involved if it was listed as a cause of death on the death certificate or in the medical examiner or coroner report. Percentages sum to >100% because drug categories are not mutually exclusive. Among the 44 jurisdictions included in demographics and circumstances analyses, a single state (West Virginia) was excluded from analyses of drug involvement, because these analyses also required jurisdictions to have data on drugs causing death for ≥75% of deaths during either 6-month reporting period. Analyses were restricted to deaths with an available medical examiner or coroner report (92.2% of all deaths included). Analyses included 62,746 total deaths (13,779 deaths among persons with any reported mental health disorder and 48,967 deaths among persons without).

^§§^ Includes heroin and 6-acetylmorphine. Morphine was coded as heroin if detected along with 6-acetylmorphine or if scene, toxicology, or witness evidence indicated presence of known heroin adulterants or impurities (including quinine, procaine, xylazine, noscapine, papaverine, thebaine, or acetylcodeine), injection, illicit drug use, or a history of heroin use.

^¶¶^ IMFs were identified using both toxicology and scene evidence because toxicology alone cannot distinguish between pharmaceutical fentanyl and IMFs.

*** Includes alfentanil, buprenorphine, butorphanol, codeine, dextrorphan, dihydrocodeine, hydrocodone, hydromorphone, levorphanol, loperamide, meperidine, methadone, morphine, nalbuphine, noscapine, oxycodone, oxymorphone, pentazocine, prescription fentanyl, propoxyphene, sufentanil, tapentadol, and tramadol. Includes brand names and metabolites (e.g., nortramadol) of these drugs and combinations of these drugs and nonopioids (e.g., acetaminophen-oxycodone). Morphine was included only if scene or witness evidence did not indicate likely heroin use and if 6-acetylmorphine was not also detected. Fentanyl was included based on scene, toxicology, or witness evidence of prescription.

^§§§^ Includes amphetamine (in the absence of methamphetamine), armodafinil, dextroamphetamine, levoamphetamine, lisdexamfetamine, mephentermine, methylphenidate, modafinil, and propylhexedrine. Includes prescription stimulant brand names and metabolites of these drugs.

### Drug Involvement

Overall, 82.2% of overdose deaths involved opioids, primarily illegally manufactured fentanyl and fentanyl analogs (75.2% of overdose deaths). Higher proportions of deaths among decedents with any MHD involved antidepressants (9.7%), benzodiazepines (15.3%), and prescription opioids (16.0%) compared with those without an MHD (3.3%, 8.5%, and 11.6%, respectively). 

### Potential Intervention Opportunities

Approximately one quarter of decedents with any reported MHD (24.5%) had one or more potential intervention opportunities in the month before death (versus 14.6% of decedents without MHD) (Figure). Decedents with reported MHD, compared with those without, more commonly experienced the following intervention opportunities: released from an institutional setting (11.2% versus 7.8%), treatment for SUD (10.1% versus 4.5%), emergency department or urgent care visit (9.5% versus 4.7%), and nonfatal overdose (4.1% versus 2.7%).

**FIGURE:**
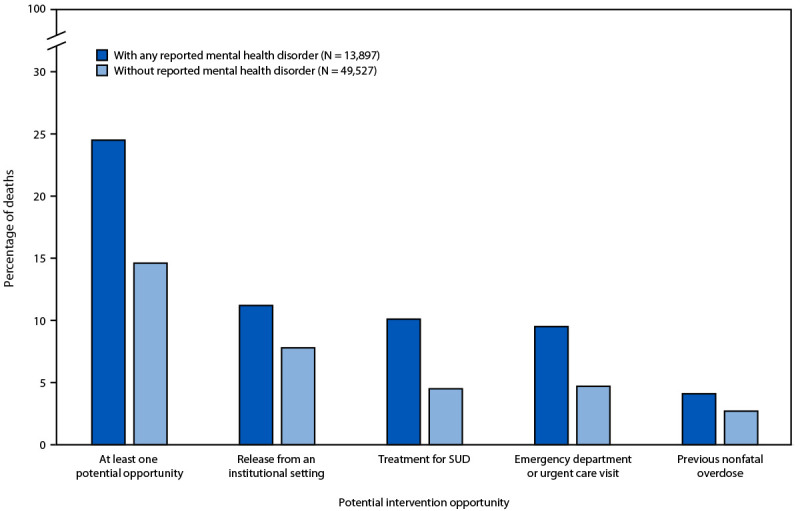
Potential opportunities for intervention* within 1 month of death among persons who died of unintentional or undetermined intent drug overdose, by non–substance-related mental health disorder status^†^ — State Unintentional Drug Overdose Reporting System, United States,^§^ 2022^¶^ **Abbreviations:** SUD = substance use disorder; SUDORS = State Unintentional Drug Overdose Reporting System. * Specific opportunities for intervention are not mutually exclusive (e.g., a person could have both current treatment for SUD and an emergency department or urgent care visit within 1 month of death and would be counted in both). Institutional setting includes prison or jail, residential treatment facility, or psychiatric hospital. ^†^ Evidence of mental health disorders was obtained from available source documents (e.g., medical records or witness report of a diagnosis in the medical examiner or coroner report) and categorized by *Diagnostic and Statistical Manual of Mental Disorders, Fifth Edition* classification. ^§^ The District of Columbia and the following 35 states reported deaths from the full jurisdiction: Alaska, Arizona, Arkansas, Colorado, Connecticut, Delaware, Georgia, Hawaii, Iowa, Kansas, Kentucky, Maine, Maryland, Massachusetts, Michigan, Minnesota, Mississippi, Montana, Nebraska, Nevada, New Hampshire, New Jersey, New Mexico, North Carolina, Ohio, Oklahoma, Oregon, Rhode Island, South Dakota, Tennessee, Utah, Vermont, Virginia, West Virginia, and Wisconsin. The following eight states reported deaths from counties that accounted for ≥75% of drug overdose deaths in the respective state, per SUDORS funding requirements: Alabama, Illinois, Indiana, Louisiana, Missouri, New York, Pennsylvania, and Washington. These 44 jurisdictions were included because death certificates and medical examiner or coroner reports were available for ≥75% of deaths during either 6-month reporting period (January–June or July–December 2022). Analyses were restricted to deaths with an available medical examiner or coroner report (92.3% of all deaths included). ^¶^ Results for all Pearson chi-square tests were p<0.05, indicating statistically significant differences for all presented results between decedents with and without a mental health disorder.

## Discussion

More than one in five persons (21.9%) who died of drug overdose in 2022 had any reported non–substance-related MHD, underscoring the importance of addressing mental health in overdose prevention and response efforts. MHDs and SUDs frequently co-occur and have shared risk factors and bidirectional associations (e.g., persons with certain MHDs might use substances to cope with their symptoms, and persons with SUDs might be at greater risk for other MHDs) (*2*,*3*). This finding suggests the need to screen for SUDs and other MHDs, which is consistent with U.S. Preventive Services Task Force (USPSTF) recommendations for adults in primary care settings,^§§§^ and the need to link and integrate treatments to prevent overdose and improve mental health (*2*).

Compared with decedents without any reported MHD, decedents with MHD were more commonly female and White, and less frequently Black and Hispanic. These sex and racial and ethnic differences could partly reflect disparities in mental health diagnoses. Historically, for example, women have been more likely to seek mental health care than men (*4*), and stigma surrounding seeking mental health care might be more pronounced among Black communities (*5*); potential racial and ethnic biases in provider diagnosing might also exist (*6*). Comprehensive screening for comorbid conditions across all demographic characteristics could decrease stigma and bias surrounding mental health and substance use and increase diagnosis and linkage to evidence-based treatment and care. For example, USPSTF recommends screening for unhealthy drug use, anxiety disorders, and depression among adults in primary care settings.^¶¶¶^

Compared with decedents without an MHD, decedents with an MHD more commonly had a known history of opioid use or misuse, and deaths in which the decedent had an MHD more often involved antidepressants, benzodiazepines, and prescription opioids. Screening for opioid use disorder and other SUDs, when persons receive a diagnosis of MHD, and screening for MHD and SUD when opioids and other drugs (e.g., antidepressants and benzodiazepines) are prescribed, could help identify co-occurring disorders and aid linkage to care (*7*). Although most overdose deaths involved opioids, it might also be helpful for providers to consider overdose risk when prescribing antidepressants and benzodiazepines among patients with a known or suspected SUD (*8*).

Approximately one quarter of decedents with an MHD had at least one potential intervention opportunity within 1 month of death; each of these reflects a possible missed opportunity to implement overdose prevention. As these touchpoint locations included emergency departments and urgent care facilities, institutions (e.g., prisons or jails and residential treatment facilities), and SUD treatment settings, the availability and expansion of substance use screening, treatment, referrals or linkage, and harm reduction services within those settings could be explored. For example, efforts to link persons with SUD to treatment services upon release from jail via peer navigators have resulted in persons expressing a desire to start or continue treatment for SUD or MHD (*9*). Further, the findings that approximately one in 10 decedents with an MHD were being treated for SUD at the time of death and one in 25 decedents with an MHD had experienced a nonfatal overdose within 1 month of death reflect important missed opportunities for prevention among persons with a high risk for overdose. This finding emphasizes the need to strengthen care integration among persons with MHD and SUD and to ensure harm reduction and linkage to treatment and care services are provided during overdose response.

### Limitations

The findings in this report are subject to at least six limitations. First, analyses include 43 or 44 jurisdictions and data for some or all of 2022 and, therefore, might not be generalizable. Second, MHD might be undiagnosed. Third, MHD diagnoses are likely underestimated because data were limited to available source documents of varying completeness. The actual percentage of decedents with MHD is likely higher than what is captured in SUDORS because of undiagnosed MHD and underestimation in source documents. Underestimation might also vary by decedent demographics. Fourth, data for current or recent mental health treatment were not available. Fifth, SUD might have been captured as an MHD when the MHD was unspecified. Finally, an MHD might not reflect a medical diagnosis consistent with DSM-5 criteria when obtained from nonmedical sources (e.g., witness reports).

### Implications for Public Health Practice

Mental health is an important consideration for drug overdose risk, and screening and integration of mental health and substance use treatment services might improve outcomes among persons with comorbid diagnoses (*10*). Adopting a multidisciplinary approach by incorporating evidence-based mental health screening into nonfatal overdose encounters (e.g., at emergency departments) and linking patients to comprehensive treatment and harm reduction services as needed might reduce overdoses and improve mental health. Persons with SUD and MHD can experience similar barriers, such as stigma, access to care, and economic factors, which could affect the willingness or ability of those facing such obstacles to seek care; removing these barriers could help reduce overdose deaths. Although SUD is a mental health disorder, some providers might experience discomfort in addressing MHD with persons who have an SUD. Therefore, provider education and training are important for addressing barriers to providing comprehensive care to persons with SUD and MHD. It is important for providers to 1) conduct evidence-based mental health screenings with persons using drugs; 2) consider overdose risk and MHD when prescribing opioids, antidepressants, and benzodiazepines, particularly among patients with known or suspected SUD; and 3) link and retain persons with SUD and MHD to treatment and harm reduction services as needed. Adopting these strategies might help prevent future overdose deaths and improve mental health.

## Footnotes

*
https://www.cdc.gov/nchs/data/databriefs/db491.pdf

†
https://nida.nih.gov/publications/research-reports/common-comorbidities-substance-use-disorders/why-there-comorbidity-between-substance-use-disorders-mental-illnesses

§
https://www.samhsa.gov/data/report/2022-nsduh-annual-national-report

¶ https://www.cdc.gov/overdose-prevention/php/od2a/index.html; https://www.cdc.gov/overdose-prevention/data-research/facts-stats/about-sudors.html

** Includes attention-deficit/hyperactivity disorder, anxiety disorder, autism spectrum, bipolar disorder, dementia (e.g., Alzheimer disease), depression or dysthymia, eating disorder, obsessive-compulsive disorder, posttraumatic stress disorder, schizophrenia, other, not applicable, and unknown. Substance-related mental health disorders, including substance use disorders and substance-induced disorders (e.g., intoxication, withdrawal, and other substance-induced mental disorders, such as substance-induced psychotic disorder), were excluded from the MHD group for this analysis because they are likely to be overrepresented among persons who died of drug overdose.

†† If a decedent had multiple disorders or “other” was selected, jurisdictions used the free text box to enter additional information. In addition, text narratives describing each death were reviewed in duplicate for instances where “unknown” or “not applicable” was selected.

§§ The District of Columbia and the following 35 states reported deaths from the full jurisdiction: Alaska, Arizona, Arkansas, Colorado, Connecticut, Delaware, Georgia, Hawaii, Iowa, Kansas, Kentucky, Maine, Maryland, Massachusetts, Michigan, Minnesota, Mississippi, Montana, Nebraska, Nevada, New Hampshire, New Jersey, New Mexico, North Carolina, Ohio, Oklahoma, Oregon, Rhode Island, South Dakota, Tennessee, Utah, Vermont, Virginia, West Virginia, and Wisconsin. The following eight states reported deaths from counties that accounted for ≥75% of drug overdose deaths in the respective state, per SUDORS funding requirements: Alabama, Illinois, Indiana, Louisiana, Missouri, New York, Pennsylvania, and Washington.

¶¶ Forty-four jurisdictions were included because death certificates and medical examiner or coroner reports were available for ≥75% of deaths during either 6-month reporting period (January–June or July–December 2022). Thirty-six jurisdictions reported data for both 6-month periods, and eight reported data for just one 6-month period (Alabama, Michigan, Nevada, New Mexico, New York, South Dakota, West Virginia, and Wisconsin). Analyses were restricted to deaths with an available medical examiner or coroner report (63,424; 92.3% of all deaths in the included jurisdictions for the first half or second half of 2022, or both).

*** Among the 44 jurisdictions included in demographics and circumstances analyses, a single state (West Virginia) was excluded from analyses of drug involvement, because these analyses also required jurisdictions to have data on drugs causing death for ≥75% of deaths during either 6-month reporting period. Analyses were restricted to deaths with an available medical examiner or coroner report (62,746; 92.2% of all deaths).

††† 45 C.F.R. part 46.102(l)(2), 21 C.F.R. part 56; 42 U.S.C. Sect. 241(d); 5 U.S.C. Sect. 552a; 44 U.S.C. Sect. 3501 et seq.

§§§
https://www.uspreventiveservicestaskforce.org/files/uspstf-2023-year-in-review.pdf

¶¶¶ https://www.uspreventiveservicestaskforce.org/uspstf/recommendation/drug-use-illicit-screening; https://www.uspreventiveservicestaskforce.org/uspstf/recommendation/anxiety-adults-screening; https://www.uspreventiveservicestaskforce.org/uspstf/recommendation/screening-depression-suicide-risk-adults
